# Supplementary material for: Structural Disconnections Caused by White Matter Hyperintensities in Post‐Stroke Spatial Neglect
Source: Hum Brain Mapp. 2024 Nov 25;45(17):e70078. doi: 10.1002/hbm.70078 (PMC11586781; doi:10.1002/hbm.70078)
Supplement: Supplementary file 1 — Data S1 Supporting Information. [file HBM-45-e70078-s001.pdf]

Supplementary Material

Structural disconnections caused by white matter hyperintensities in post-stroke spatial neglect

Lisa Röhrig[1] and Hans-Otto Karnath[1]

[1] Center of Neurology, Division of Neuropsychology, Hertie Institute for Clinical Brain Research, University of Tübingen, 72076 Tübingen, Germany

Supplementary Analyses

Analysis of bilateral white matter hyperintensities

A recent simulation study by Röhrig et al. (2023) found that for unilateral brain damage (either in the left or right hemisphere), structural disconnections should be analyzed separately for each hemisphere. Combining them in a joint analysis can lead to a statistical imbalance, which disadvantages intra-hemispheric disconnections (Röhrig et al., 2023). In present analyses, we investigated patients with unilateral stroke damage and bilateral white matter hyperintensities (WMH). Since bilateral WMH affect both hemispheres simultaneously (despite some asymmetries), each type of disconnection can potentially occur in each individual (i.e. inter-hemispheric disconnections just as left- and right-sided intra-hemispheric disconnections). This circumstance results in inter- and intra-hemispheric disconnections being balanced when jointly analyzing left and right hemispheric WMH, indicating the unnecessity for separating bilateral WMH-related structural disconnections by hemisphere. For this reason, we here present joint analyses of bilateral WMH. Analyses using only right-sided WMH, however, are presented within the main manuscript; it was previously shown that left-sided WMH were less informative than right-sided WMH, and right-sided and bilateral WMH were almost equally predictive (Röhrig et al., 2022).

Voxel-wise results

When analyzing voxel-wise disconnectivity derived from the joint topography of stroke lesion and bilateral WMH, 221 voxels were significantly associated with neglect severity ($z > 5.93$). The voxel with the highest *z*-score of 6.84 is part of the right inferior frontal junction within the MFG (MNI-coordinates = 39, 8, 47). Among the white matter tracts, significant voxels were most frequently part of the right fronto-pontine tract, right cortico-striatal pathway, and right U-fibers (HCP-842; Yeh et al., 2018); significant voxels further cover white matter underlying the right SFG, MFG, and basal ganglia (BNA; Fan et al., 2016). We found 77 voxels to be more strongly associated to neglect severity when bilateral WMH were considered; the voxel with the largest increase is located at the right cortico-thalamic pathway next to the dorsal caudate (MNI-coordinates = 20, 2, 20).

Pairwise results

When combining stroke lesion and bilateral WMH, we identified significant disconnections: $N = 62$ at $p < 0.05$ ($t \geq 4.59$); $N = 14$ at $p < 0.01$ ($t \geq 5.38$); $N = 5$ at $p < 0.005$ ($t \geq 5.72$). In total, 31 gray matter regions were affected. The right globus pallidus was the only gray matter region with at least 10 disconnections. The most significant disconnections were found within the right hemisphere between the ventrolateral area 6 of the MFG and globus pallidus respectively dorsolateral putamen respectively posterior parietal thalamus, between globus pallidus and inferior frontal junction of the MFG, and between the dorsolateral area 8 of the SFG and dorsal area 9/46 of the MFG. We found 41 pairwise connections with a stronger association to neglect behavior when bilateral WMH were involved; the pairwise connection with the largest increase connects the right ventrolateral area 8 of the MFG and the right posterior parietal thalamus.

Tract-wise results

The joint topography of stroke lesion and bilateral WMH did reveal tracts significantly related to neglect severity: $N = 11$ at $p < 0.05$ ($t > 3.44$); $N = 7$ at $p < 0.01$ ($t > 4.29$); $N = 5$ at $p < 0.005$ ($t > 4.63$); $N = 3$ at $p < 0.001$ ($t > 5.34$). Among most significant tracts, we found the right U-fibers, right cortico-striatal pathway, and right fronto-pontine tract to be highly associated with neglect severity. We found 4 white matter tracts with a stronger association to neglect behavior when bilateral WMH were considered; the right fronto-pontine tract received the largest increase in association strength.

Parcel-wise results

When bilateral WMH were combined with the individual stroke lesion, findings revealed gray matter parcels significantly related to neglect severity: $N = 9$ at $p < 0.05$ ($t > 4.76$); $N = 3$ at $p < 0.01$ ($t > 5.52$); $N = 1$ at $p < 0.005$ ($t > 5.87$). The most significant parcel was rostral area 21 of the right MTG. We found no parcel that was stronger associated to neglect behavior when bilateral WMH were considered.

Supplementary Tables

Table S1. Number of features used for statistical analyses.

Disconnection origin	Disconnection type			
	Voxel-wise	Pairwise	Tract-wise	Parcel-wise
Stroke lesion	330,557	2024	30	80
Right-sided WMH	305,699	2065	27	39
Bilateral WMH	483,673	2818	49	83
Stroke lesion + right-sided WMH	389,934	2177	31	102
Stroke lesion + bilateral WMH	559,905	2919	52	145

Note. To ignore features that were not at all or less frequently damaged (i.e. features with no or little variance), we restricted our main analyses (GLMs and prediction analyses) to features that were affected in a minimum number of patients. For voxel-wise data, we ignored voxels damaged in less than 5 patients, whereas for pairwise, tract-wise, and parcel-wise data, we removed features damaged in less than 10 patients. Total number of features before restriction: 985,860 voxels, 60,516 pairwise connections, 70 white matter tracts, and 246 gray matter parcels. Principal component analysis was performed on voxel-wise data after feature restriction for predictive modeling: for stroke lesion-induced disconnectivity, we kept 76 components cumulatively explaining > 99.5 % of the imaging variance; for right-sided WMH-induced disconnectivity, we kept 38 components cumulatively explaining > 90 % of the imaging variance; for bilateral WMH-induced disconnectivity, we kept 44 components cumulatively explaining > 90 % of the imaging variance; for stroke lesion and right-sided WMH-induced disconnectivity, we kept 39 components explaining > 90 % variance; for stroke lesion and bilateral WMH-induced disconnectivity, we kept 96 components explaining > 99.5 % variance (i.e. model-specific optima).

Table S2. Most significant results of pairwise analyses.

Stroke lesion			Stroke lesion + right WMH			Stroke lesion + bilateral WMH		
Region A<br>*Region B*	*t*	*p(FWE)*	*Region A*<br>*Region B*	*t*	*p(FWE)*	*Region A*<br>*Region B*	*t*	*p(FWE)*
MFG_R_A6vl<br>IFG_L_A44op	7.123	< 0.001	MFG_ R_A6vl<br>BG_R_GP	6.005	< 0.001	MFG_ R_A6vl<br>BG_R_GP	6.005	< 0.005
MFG_R_A8vl<br>IFG_L_A44op	6.698	< 0.001	MFG_R_IFJ<br>BG_R_GP	5.990	< 0.001	MFG_R_IFJ<br>BG_R_GP	5.990	< 0.005
MFG_R_IFJ<br>Tha_R_PPtha	6.479	< 0.005	MFG_ R_A6vl<br>BG_R_dlPu	5.856	< 0.005	MFG_ R_A6vl<br>BG_R_dlPu	5.856	< 0.005
MFG_R_A8vl<br>INS_L_dIa	6.453	< 0.005	MFG_ R_A6vl<br>Tha_R_PPtha	5.851	< 0.005	MFG_ R_A6vl<br>Tha_R_PPtha	5.851	< 0.005
MFG_ R_A8vl<br>IFG_L_A44v	6.451	< 0.005	SFG_R_A8dl<br>MFG_R_A9/46d	5.735	< 0.005	SFG_R_A8dl<br>MFG_R_A9/46d	5.735	< 0.005
MFG_R_IFJ<br>BG_R_dCa	6.372	< 0.005	MFG_R_IFJ<br>BG_R_dlPu	5.715	< 0.005	MFG_R_IFJ<br>BG_R_dlPu	5.715	< 0.01
MFG_ R_A6vl<br>IFG_L_A44v	6.309	< 0.005	MFG_ R_A6vl<br>Tha_R_cTtha	5.632	< 0.005	MFG_ R_A6vl<br>Tha_R_cTtha	5.632	< 0.01
MFG_L_A8vl<br>MFG_ R_A6vl	6.265	< 0.005	MFG_ R_A6vl<br>Tha_R_mPMtha	5.626	< 0.005	MFG_ R_A6vl<br>Tha_R_mPMtha	5.626	< 0.01
MFG_R_IFJ<br>IPL_R_A39rd	6.179	< 0.005	SFG_R_A8dl<br>BG_R_GP	5.565	< 0.005	SFG_R_A8dl<br>BG_R_GP	5.565	< 0.01
MFG_L_A6vl<br>MFG_ R_A6vl	6.088	< 0.005	MFG_ R_A6vl<br>Tha_R_lPFtha	5.541	< 0.005	MFG_ R_A6vl<br>Tha_R_lPFtha	5.541	< 0.01
MFG_ R_A6vl<br>INS_L_vIa	6.034	< 0.005	MFG_R_IFJ<br>Tha_R_mPMtha	5.502	< 0.005	MFG_R_IFJ<br>Tha_R_mPMtha	5.502	< 0.01
MFG_R_A9/46d<br>MFG_L_A8vl	5.966	< 0.005	SFG_R_A6dl<br>BG_R_dlPu	5.416	< 0.005	SFG_R_A6dl<br>BG_R_dlPu	5.416	< 0.01
STG_R_A38l<br>IPL_L_A39rd	5.946	< 0.005	MTG_L_A21r<br>PhG_R_TI	5.414	< 0.005	STG_R_A38m<br>INS_R_vIa	5.398	< 0.01
MTG_R_A21r<br>LOcC_R_msOccG	5.879	< 0.005	STG_R_A38m<br>INS_R_vIa	5.398	< 0.005	SFG_R_A8dl<br>Tha_R_PPtha	5.393	< 0.01
MFG_ R_A8vl<br>INS_L_dIg	5.877	< 0.005	SFG_R_A8dl<br>Tha_R_PPtha	5.393	< 0.005	MFG_ R_A8vl<br>BG_R_GP	5.356	< 0.05
MFG_R_IFJ<br>MFG_L_A6vl	5.874	< 0.005	MFG_ R_A8vl<br>BG_R_GP	5.356	< 0.005	IFG_R_A44d<br>Tha_R_lPFtha	5.278	< 0.05
MFG_ R_A6vl<br>INS_L_dIa	5.868	< 0.005	IFG_R_A44d<br>Tha_R_lPFtha	5.278	< 0.005	MFG_ R_A8vl<br>Tha_R_mPMtha	5.220	< 0.05
MFG_ R_A6vl<br>BG_R_dCa	5.857	< 0.005	MFG_ R_A6vl<br>IFG_L_A44v	5.275	< 0.005	SFG_R_A8dl<br>Tha_R_lPFtha	5.194	< 0.05
STG_R_A38l<br>MTG_L_A21r	5.855	< 0.005	MFG_ R_A8vl<br>Tha_R_mPMtha	5.220	< 0.01	IFG_R_A44d<br>Tha_R_mPMtha	5.193	< 0.05
STG_R_A38l<br>ITG_L_A20cl	5.852	< 0.005	SFG_R_A8dl<br>Tha_R_lPFtha	5.194	< 0.01	SFG_R_A6m<br>BG_R_dlPu	5.192	< 0.05
STG_R_A38l<br>ITG_L_A37elv	5.839	< 0.005	IFG_R_A44d<br>Tha_R_mPMtha	5.193	< 0.01	SFG_R_A8dl<br>Tha_R_mPMtha	5.190	< 0.05
MFG_R_IFJ<br>BG_R_dlPu	5.834	< 0.005	SFG_R_A6m<br>BG_R_dlPu	5.192	< 0.01	SFG_R_A8m<br>BG_R_dlPu	5.170	< 0.05
MTG_L_A21r<br>MTG_R_A21r	5.831	< 0.005	SFG_R_A8dl<br>Tha_R_mPMtha	5.190	< 0.01	MFG_ R_A6vl<br>Tha_R_Otha	5.164	< 0.05
MFG_R_IFJ<br>Tha_R_lPFtha	5.805	< 0.005	SFG_R_A8m<br>BG_R_dlPu	5.170	< 0.01	SFG_R_A8dl<br>BG_R_dlPu	5.163	< 0.05
STG_R_A38l<br>LOcC_L_V5/MT+	5.795	< 0.005	MFG_ R_A6vl<br>Tha_R_Otha	5.164	< 0.01	MFG_ R_A8vl<br>Tha_R_PPtha	5.082	< 0.05

Note. Table reports 25 region pairs most significantly associated to neglect severity, sorted by decreasing *t*-statistics. Results were obtained from GLMs using pairwise structural disconnection data. *P*-thresholds were obtained using permutation-based FWE-correction. Disconnections were derived by either stroke lesions or stroke lesions and right-sided WMH combined. Total number of significant pairwise disconnections: *N(stroke lesion)* = 394, *N(stroke lesion and right WMH)* = 112, *N(stroke lesion and bilateral WMH)* = 62. Parcels and respective abbreviations are based on the Brainnetome Atlas with 246 regions (Fan et al., 2016).

Abbreviations: BG – basal ganglia, IFG – inferior frontal gyrus, INS – insular gyrus, IPL – inferior parietal lobe, ITG – inferior temporal gyrus, L – left hemisphere, LOcC – lateral occipital cortex, MFG – middle frontal gyrus, MTG – middle temporal gyrus, PhG – parahippocampal gyrus, R – right hemisphere, SFG – superior frontal gyrus, STG – superior temporal gyrus, Tha - thalamus.

Table S3. Region counts of pairwise disconnections.

Stroke lesion		Stroke lesion + right WMH		Stroke lesion + bilateral WMH	
Region	*Count*	*Region*	*Count*	*Region*	*Count*
STG_R_A38l	28	MFG_R_A6vl	15	BG_R_GP	10
MFG_R_A6vl	26	BG_R_GP	15		
ITG_R_A37vl	19	BG_R_dlPu	15		
MFG_R_IFJ	18	SFG_R_A8dl	11		
MFG_R_A8vl	18	MFG_R_A8vl	11		
PrG_R_A4tl	18	Tha_R_mPMtha	10		
MTG_R_A37dl	18				
IFG_R_A44v	17				
PrG_R_A4hf	16				
ITG_R_A37elv	16				
ITG_R_A20iv	15				
ITG_R_A20cl	15				
MTG_R_A21r	14				
IFG_R_A44d	12				
MTG_L_A21r	12				
INS_R_dId	12				
BG_R_GP	12				
BG_R_dlPu	12				
SFG_R_A8dl	11				
IPL_L_A39c	11				
IPL_R_A39rd	11				
IPL_R_A40rd	11				

Note. Table reports all regions that were involved in at least 10 pairwise structural disconnections significantly associated to neglect severity ($p < 0.05$, with permutation-based FWE-correction). Disconnections were derived by either stroke lesions or stroke lesions and right-sided WMH combined. Parcels and respective abbreviations are based on the Brainnetome Atlas with 246 regions (Fan et al., 2016). For visualization, see Figure S1.

Abbreviations: BG – basal ganglia, IFG – inferior frontal gyrus, INS – insular gyrus, IPL – inferior parietal lobe, ITG – inferior temporal gyrus, L – left hemisphere, MFG – middle frontal gyrus, MTG – middle temporal gyrus, PrG – precentral gyrus, R – right hemisphere, SFG – superior frontal gyrus, STG – superior temporal gyrus, Tha - thalamus.

Table S4. Significant results of tract-wise analyses.

Stroke lesion			Stroke lesion + right WMH			Stroke lesion + bilateral WMH		
Tract	*t*	*p(FWE)*	*Tract*	*t*	*p(FWE)*	*Tract*	*t*	*p(FWE)*
U_R	7.551	< 0.001	U_R	5.973	< 0.001	U_R	5.973	< 0.001
SLF_R	6.158	< 0.001	CS_R	5.358	< 0.001	CS_R	5.358	< 0.001
CT_R	5.823	< 0.001	FPT_R	5.339	< 0.001	FPT_R	5.339	< 0.001
CCMidAnterior	5.597	< 0.001	CT_R	5.174	< 0.001	CT_R	5.174	< 0.005
CS_R	5.467	< 0.001	SLF_R	4.750	< 0.005	SLF_R	4.750	< 0.005
CCCentral	5.204	< 0.001	UF_R	4.423	< 0.005	UF_R	4.423	< 0.01
AF_R	4.963	< 0.005	CST_R	4.346	< 0.005	CST_R	4.346	< 0.01
FPT_R	4.889	< 0.005	PPT_R	4.192	< 0.005	PPT_R	4.192	< 0.05
CCPosterior	4.880	< 0.005	AC	4.152	< 0.01	AC	4.168	< 0.05
CCAnterior	4.876	< 0.005	CCMidAnterior	4.050	< 0.01	FAT_R	4.011	< 0.05
FAT_R	4.594	< 0.005	FAT_R	4.011	< 0.01	EMC_R	3.718	< 0.05
EMC_R	4.422	< 0.005	EMC_R	3.718	< 0.05			
CCMidPosterior	4.359	< 0.005	CCCentral	3.424	< 0.05			
UF_R	4.255	< 0.01	AF_R	3.253	< 0.05			
AR_R	4.239	< 0.01						
AC	4.231	< 0.01						
CST_R	4.128	< 0.01						
TPT_R	3.996	< 0.05						
PPT_R	3.915	< 0.05						
OPT_R	3.874	< 0.05						
MdLF_R	3.630	< 0.05						
IFOF_R	3.313	< 0.05						

Note. Table reports all white matter tracts that were significantly associated to neglect severity, sorted by decreasing *t*-statistics. Results were obtained from GLMs using tract-wise structural disconnection data. Disconnections were derived by either stroke lesions or stroke lesions and right-sided WMH combined. *P*-thresholds were obtained using permutation-based FWE-correction. Tracts and respective abbreviations are based on the HCP-842 tractography atlas (Yeh et al., 2018) and the segmentation of the corpus callosum (FreeSurferSeg ROIs distributed with DSI Studio, https://dsi-studio.labsolver.org/) with total 70 tracts.

Abbreviations: AC – anterior commissure, AF – arcuate fasciculus, AR – acoustic radiation, CC – corpus callosum, CS – cortico-striatal pathway, CST – cortico-spinal tract, CT – cortico-thalamic pathway, EMC – extreme capsule, FAT – frontal-aslant tract, FPT – fronto-pontine tract, IFOF – inferior fronto-occipital

fasciculus, MdLF – middle longitudinal fasciculus, OPT – occipito-pontine tract, PPT – parieto-pontine tract, R – right hemisphere, SLF – superior longitudinal fasciculus, TPT- temporo-pontine tract, U – U-fiber, UF – uncinate fasciculus.

Table S5. Significant results of parcel-wise analyses.

Stroke lesion			Stroke lesion + right WMH			Stroke lesion + bilateral WMH		
Parcel	*t*	*p(FWE)*	*Parcel*	*t*	*p(FWE)*	*Parcel*	*t*	*p(FWE)*
MTG_A21r	6.198	< 0.005	MTG_A21r	6.197	< 0.005	MTG_A21r	6.197	< 0.005
STG_A38m	5.752	< 0.005	STG_A38m	5.752	< 0.005	STG_A38m	5.752	< 0.01
PrG_A6cvl	5.628	< 0.005	PrG_A6cvl	5.608	< 0.01	PrG_A6cvl	5.608	< 0.01
STG_A38l	5.313	< 0.01	STG_A38l	5.311	< 0.01	STG_A38l	5.311	< 0.05
MFG_IFJ	5.288	< 0.01	MFG_IFJ	5.284	< 0.05	MFG_IFJ	5.284	< 0.05
PrG_A4tl	5.226	< 0.05	PrG_A4tl	5.222	< 0.05	PrG_A4tl	5.222	< 0.05
MFG_A6vl	5.063	< 0.05	MFG_A6vl	5.035	< 0.05	MFG_A6vl	5.035	< 0.05
INS_vId/vIg	4.954	< 0.05	INS_vId/vIg	4.954	< 0.05	INS_vId/vIg	4.954	< 0.05
IFG_A44v	4.798	< 0.05	IFG_A44v	4.791	< 0.05	IFG_A44v	4.791	< 0.05
IPL_A39c	4.728	< 0.05	IPL_A39c	4.738	< 0.05			
STG_TE1.0/.2	4.492	< 0.05						
OrG_A11l	4.445	< 0.05						
BG_GP	4.404	< 0.05						

Note. Table reports all parcels that were significantly associated to neglect severity, sorted by decreasing *t*-statistics. Results were obtained from GLMs using parcel-wise structural disconnection data. Disconnections were derived by either stroke lesions or stroke lesions and right-sided WMH combined. *P*-thresholds were obtained using permutation-based FWE-correction. Parcels and respective abbreviations are based on the Brainnetome Atlas with 246 regions (Fan et al., 2016). All reported parcels are right hemispheric. For visualization, see Figure S2.

Abbreviations: BG – basal ganglia, IFG – inferior frontal gyrus, INS – insular gyrus, IPL – inferior parietal lobe, MFG – middle frontal gyrus, MTG – middle temporal gyrus, OrG – orbital gyrus, PrG – precentral gyrus, STG – superior temporal gyrus.

Table S6. WMH-related increase in statistical association strength.

Pairwise				Tract-wise			
Region A<br>*Region B*	*t*<br>*(stroke)*	*t (stroke + WMH)*	Δ*t*	*Tract*	*t*<br>*(stroke)*	*t (stroke + WMH)*	Δ*t*
MFG_R_A8vl<br>Tha_R_PPtha	4.209	5.082	0.873	FPT_R	4.889	5.339	0.450
PrG_R_A4t<br>BG_R_GP	3.641	4.489	0.849	PPT_R	3.915	4.192	0.277
PCL_R_A4ll<br>BG_R_dlPu	3.575	4.421	0.847	CST_R	4.128	4.346	0.217
SFG_R_A8dl<br>Tha_R_mPMtha	4.348	5.190	0.842	UF_R	4.255	4.423	0.168
SFG_R_A8dl<br>PrG_R_A6cdl	3.432	4.224	0.793				
MFG_R_A9/46d<br>Tha_R_rTtha	4.254	5.014	0.760				
SFG_R_A8dl<br>BG_R_GP	4.828	5.565	0.736				
SFG_R_A6dl<br>Tha_R_mPMtha	4.109	4.832	0.723				
IFG_R_A44d<br>Tha_R_lPFtha	4.564	5.278	0.714				
SFG_R_A8m<br>Tha_R_mPMtha	4.091	4.799	0.708				

Note. Table reports pairwise connections and white matter tracts with a stronger association to neglect severity when right-sided WMH were considered. For pairwise results, top 10 connections with the largest increase are presented (from a total of 56 connections). To identify connections/tracts that were more strongly associated with neglect severity due to WMH involvement, we only considered connections/tracts that were tested in both analyses (stroke lesion with and without WMH), that resulted in $\Delta t > 0.01$, and that reached significance when WMH were considered ($p < 0.05$). Parcels and respective abbreviations are based on the Brainnetome Atlas with 246 regions (Fan et al., 2016). Tracts and respective abbreviations are based on the HCP-842 tractography atlas (Yeh et al., 2018) and the segmentation of the corpus callosum (FreeSurferSeg ROIs distributed with DSI Studio, https://dsi-studio.labsolver.org/) with total 70 tracts.

Abbreviations: BG – basal ganglia, CST – cortico-spinal tract, FPT – fronto-pontine tract, IFG – inferior frontal gyrus, MFG – middle frontal gyrus, PCL – paracentral lobule, PPT – parieto-pontine tract, PrG – precentral gyrus, R – right hemispheric, SFG – superior frontal gyrus, Tha – thalamus, UF – uncinate fasciculus.

Supplementary Figures

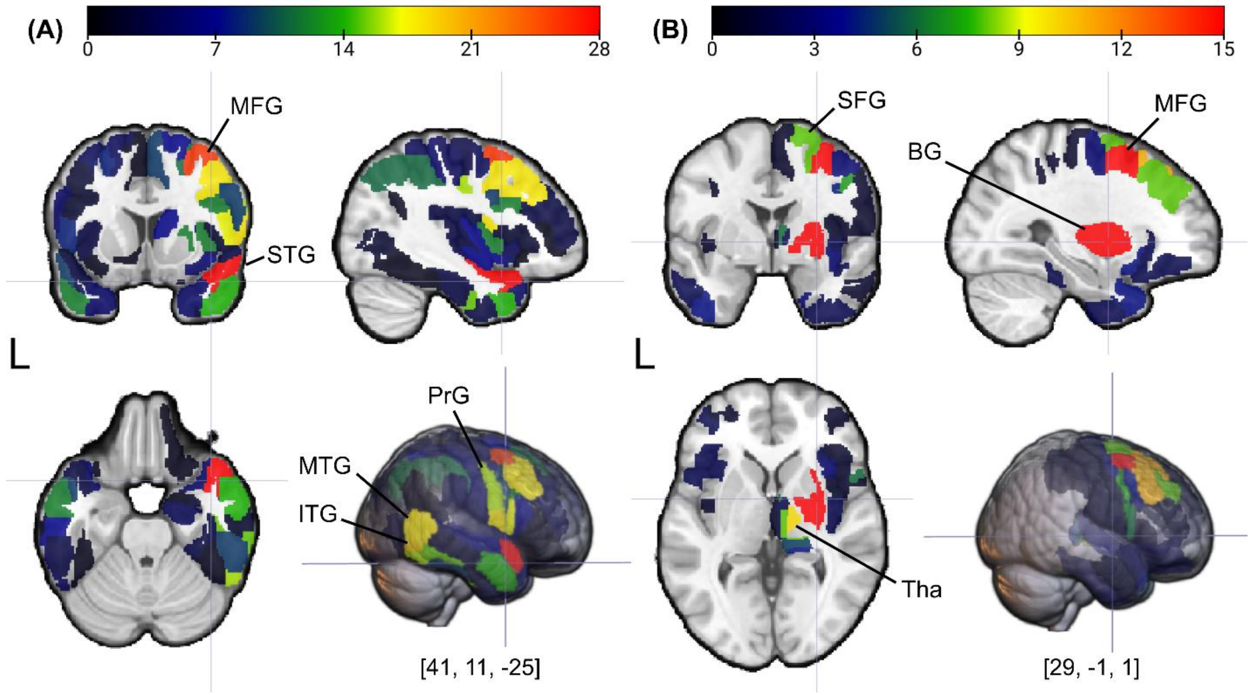

Figure S1. Brain regions most frequently involved in significant pairwise structural disconnections. Cortical and subcortical brain parcels of the Brainnetome Atlas (Yeh et al., 2018) are color-coded based on respective disconnection frequencies, i.e. how often a region was involved in region-to-region disconnections significantly associated to neglect severity (p < 0.05, with permutation-based FWE-correction). Pairwise disconnections were derived by either **(A)** stroke lesions or **(B)** stroke lesions and right-sided WMH combined. Coronal, sagittal, and axial views as well as the whole brain are presented; crosshair is located at (one of) the most frequently disconnected region (MNI coordinates are reported in square brackets). For details, see Table S3.

Abbreviations: BG – basal ganglia, ITG – inferior temporal gyrus, L – left hemisphere, MFG – middle frontal gyrus, MTG – middle temporal gyrus, PrG – precentral gyrus, SFG – superior frontal gyrus, STG – superior temporal gyrus, Tha - thalamus.

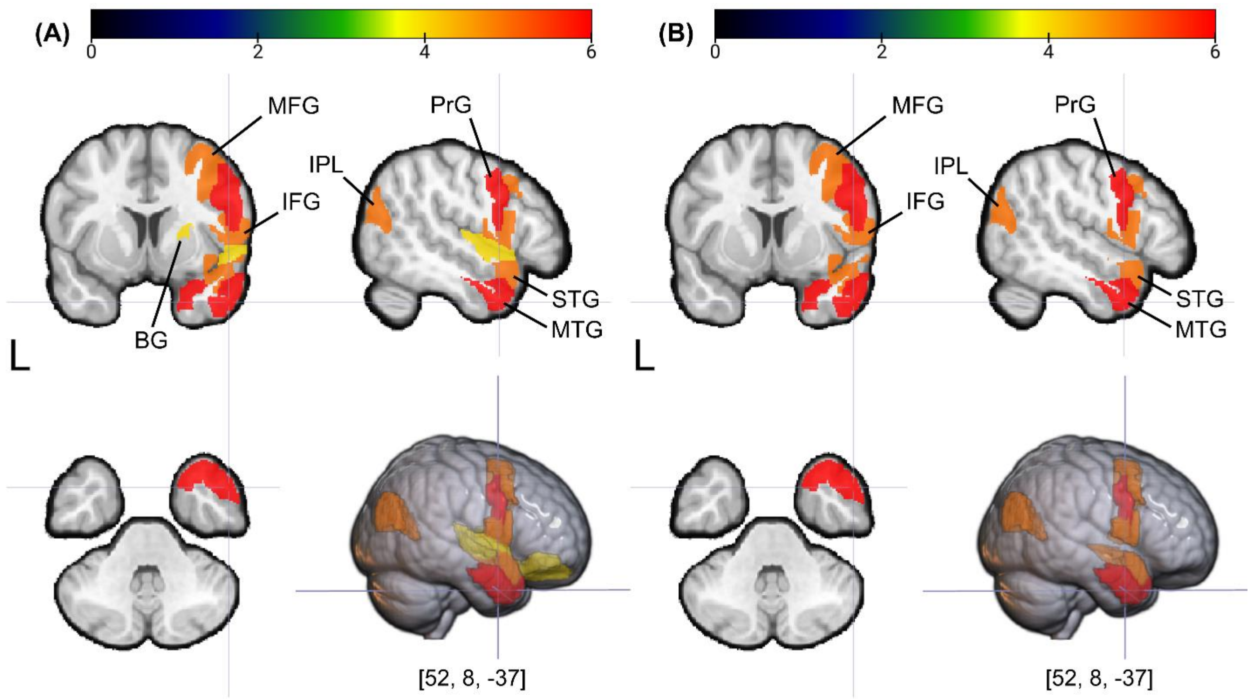

Figure S2. Parcel-wise results. Significant cortical and subcortical brain parcels of the Brainnetome Atlas (Yeh et al., 2018) are color-coded based on respective *t*-statistics, i.e. how strongly parcel-wise structural disconnection was associated to neglect severity ($p < 0.05$, with permutation-based FWE-correction). Parcel-wise disconnections were derived by either **(A)** stroke lesions or **(B)** stroke lesions and right-sided WMH combined. Coronal, sagittal, and axial views as well as the whole brain are presented; crosshair is located at the most strongly associated region (MNI coordinates are reported in square brackets). For details, see Table S5.

Abbreviations: BG – basal ganglia, IFG – inferior frontal gyrus, IPL – inferior parietal lobe, L – left hemisphere, MFG – middle frontal gyrus, MTG – middle temporal gyrus, PrG – precentral gyrus, STG – superior temporal gyrus.

References

Fan, L., Li, H., Zhuo, J., Zhang, Y., Wang, J., Chen, L., Yang, Z., Chu, C., Xie, S., Laird, A. R., Fox, P. T., Eickhoff, S. B., Yu, C., & Jiang, T. (2016). The Human Brainnetome Atlas: A New Brain Atlas Based on Connectional Architecture. *Cerebral Cortex*, *26*(8), 3508–3526. https://doi.org/10.1093/cercor/bhw157

Röhrig, L., Rosenzopf, H., Wöhrstein, S., & Karnath, H.-O. (2023). The need for hemispheric separation in pairwise structural disconnection studies. *Human Brain Mapping*, *44*(16), 5212–5220. https://doi.org/10.1002/hbm.26445

Röhrig, L., Sperber, C., Bonilha, L., Rorden, C., & Karnath, H.-O. (2022). Right hemispheric white matter hyperintensities improve the prediction of spatial neglect severity in acute stroke. *NeuroImage: Clinical*, *36*, 103265. https://doi.org/10.1016/j.nicl.2022.103265

Yeh, F.-C., Panesar, S., Fernandes, D., Meola, A., Yoshino, M., Fernandez-Miranda, J. C., Vettel, J. M., & Verstynen, T. (2018). Population-averaged atlas of the macroscale human structural connectome and its network topology. *NeuroImage*, *178*, 57–68. https://doi.org/10.1016/j.neuroimage.2018.05.027
